# Effect of tramadol as an adjuvant to local anesthetics for brachial plexus block: A systematic review and meta-analysis

**DOI:** 10.1371/journal.pone.0184649

**Published:** 2017-09-27

**Authors:** Hye Won Shin, Bum Jun Ju, Yoo Kyung Jang, Hae Seun You, Hyun Kang, Ji Yong Park

**Affiliations:** 1 Department of Anesthesiology and Pain Medicine, College of Medicine, Korea University Anam Hospital, Seoul, Republic of Korea; 2 Department of Anesthesiology and Pain Medicine, College of Medicine, Chung-Ang University Hospital, Seoul, Republic of Korea; University of Toronto, CANADA

## Abstract

**Background:**

Tramadol, a 4-phenyl-piperidine analog of codeine, has a unique action in that it has a central opioidergic, noradrenergic, serotonergic analgesic, and peripheral local anesthetic (LA) effect. Many studies have reported contradictory findings regarding the peripheral analgesic effect of tramadol as an adjuvant to LA in brachial plexus block (BPB). This meta-analysis aimed to evaluate the effects of tramadol as an adjunct to LA in BPB during shoulder or upper extremity surgery.

**Methods:**

We searched the PubMed, EMBASE, Cochrane, KoreaMed databases, and Google Scholar for eligible randomized controlled trials (RCTs) that compared BPB with LA alone and BPB with LA and tramadol. Primary outcomes were the effects of tramadol as an adjuvant on duration of sensory block, motor block, and analgesia. Secondary outcomes were the effects of tramadol as an adjuvant on time to onset of sensory block and motor block and on adverse effects. We performed the meta-analysis using Review Manager 5.3 software.

**Results:**

We identified 16 RCTs with 751 patients. BPB with tramadol prolonged the duration of sensory block (mean difference [MD], -61.5 min; 95% CI, -95.5 to -27.6; *P* = 0.0004), motor block (MD, -65.6 min; 95% CI, -101.5 to -29.7; *P* = 0.0003), and analgesia (MD, -125.5 min; 95% CI, -175.8 to -75.3; *P* < 0.0001) compared with BPB without tramadol. Tramadol also shortened the time to onset of sensory block (MD, 2.1 min; 95% CI, 1.1 to 3.1; *P* < 0.0001) and motor block (MD, 1.2 min; 95% CI, 0.2 to 2.1; *P* = 0.010). In subgroup analysis, the duration of sensory block, motor block, and analgesia was prolonged for BPB with tramadol 100 mg (*P* < 0.05) but not for BPB with tramadol 50 mg. The quality of evidence was high for duration of analgesia according to the GRADE system. Adverse effects were comparable between the studies.

**Conclusions:**

In upper extremity surgery performed under BPB, use of tramadol 100 mg as an adjuvant to LA appears to prolong the duration of sensory block, motor block, and analgesia, and shorten the time to onset of sensory and motor blocks without altering adverse effects.

## Introduction

A brachial plexus block (BPB) provides anesthesia and analgesia during surgery involving the upper limb and for acutely painful conditions, and is the most frequent plexus block performed by anesthesiologists. It is worthwhile to explore the options for extending pain relief while minimizing the adverse effects of local anesthesia. Local anesthetics (LAs) have been used with various perineural adjuvants, including dexamethasone [1, 2], clonidine [3], dexmedetomidine [4], opioids [5], and magnesium [6], to enhance the quality and duration of anesthesia and postoperative analgesia.

Systemic opioids have been used to relieve pain during surgery for many years, but the effects of perineural opioid adjuvants on BPB are controversial. Some studies have reported that addition of opioids such as fentanyl, alfentanil, morphine, buprenorphine, and meperidine to BPB improves sensory block, motor block, and analgesia, but other studies have found no such effect [7–9].

Tramadol administered parenterally or orally has proven effective in managing acute postoperative pain in adults [10]. Tramadol is a unique opioid with two modes of action for inhibition of pain, i.e., an opioid action mediated by the μ receptor and a non-opioid action mediated by α_2_-adrenergic and serotoninergic activity [11, 12]. The monoaminergic activity of tramadol inhibits the descending pain pathways, resulting in suppression of nociceptive transmission at the spinal level [13]. Tramadol also exhibits LA properties by blocking K^+^ channels [14]. Clinically, intradermal administration of tramadol provides local anesthesia for minor skin procedures [15]. Many studies have characterized the effects of tramadol as an adjuvant to LA in BPB [16–31]. However, these studies have yielded variable results regarding the analgesia-enhancing effects of tramadol when used in BPB; while some studies showed a beneficial effect, others showed no benefit.

The purpose of this meta-analysis and systematic review was to evaluate the effects of tramadol as an adjunct to LA in BPB on the onset and duration of sensory block, motor block, and analgesia, as well as the adverse effects associated with BPB when used for shoulder and upper extremity surgery.

## Materials and methods

This meta-analysis of randomized controlled trials (RCTs) evaluated the effect of tramadol as an adjuvant to LA in BPB and was performed according to the recommendations of the PRISMA statement. The systematic review was registered on PROSPERO under the number CRD42015023489.

### Literature search

Following the protocol recommended by the Cochrane Collaboration, we performed a systematic literature search for RCTs to evaluate the effects of tramadol as an adjunct to LA in BPB for shoulder or upper extremity surgery. The PubMed, EMBASE, Cochrane CENTRAL, and KoreaMed databases as well as Google Scholar were systematically searched for RCTs performed in adults (aged older than 18 y) up to November 2015 without language restrictions. The search strategy comprised the following key words: (“tramadol”) and (“local anesthetic”) and (“axillary block” or “brachial plexus block” or “infraclavicular block” or “interscalene block” or “supraclavicular block”) as outlined in Supporting Information (S1 File).

### Study selection

The studies included in this analysis were peer-reviewed RCTs that compared BPB with LA alone and BPB with LA and tramadol for shoulder or upper extremity surgery in adult patients. Review articles, case reports, letters to the editor, commentaries, proceedings, laboratory studies, and other non-relevant studies were excluded. Two authors (JBJ and YKC) independently assessed the articles for compliance with the inclusion/exclusion criteria. Any disagreement was resolved by discussion or consultation with a third independent investigator (HWS).

### Data extraction and assessment of outcomes

The primary outcomes were the effects of tramadol as an adjuvant to LA on duration of sensory block, motor block, and analgesia. The secondary outcomes were the effects of tramadol as an adjuvant to LA on time to onset of motor block and sensory block and on the adverse effects of BPB for shoulder and upper extremity surgery.

Using standardized forms, two authors (JBJ and JYP) independently extracted the following data: the name of the first author, year of publication, type of surgery, type and dose of LA, volume of LA, dose of tramadol, number of patients, technique used for nerve guidance (landmark, nerve stimulator, or ultrasound guidance), type of BPB approach (axillary, infraclavicular, interscalene, or supraclavicular), definitions of sensory and motor block (duration of sensory block, duration of motor block, duration of analgesia, onset of sensory block, and onset of motor block), and adverse effects (nausea, vomiting, pruritus, and sedation). In our analysis, there were two studies that contained more than two groups for tramadol as an adjuvant to LA (one by Kabachi et al.[24] that included arms receiving tramadol 100 mg and 200 mg and the other by Robaux et al.[29] that included arms receiving tramadol 40 mg, 100 mg, and 200 mg). Data from RCTs with more than two intervention groups need to be combined into a single group according to the formula for combining groups in the Cochrane Handbook [32]. However, we used only the data for the 50 mg and 100 mg doses in the meta-analysis for comparison of the effects of tramadol according to dose strength. We attempted to contact the authors of studies that had insufficient or missing data. If contact was not possible, we extrapolated data from the study text or tables to obtain the relevant information. Values for the duration and time to onset of sensory or motor block were converted into minutes and the adverse effects of BPB were reported as the number of patients. The control group included patients who received LA alone in BPB and the intervention group included those who received LA with tramadol in BPB during surgery.

### Assessment of risk of bias

Two authors (JBJ and YKJ) independently evaluated the quality of the RCTs by using the risk of bias tool in Review Manager (RevMan 5.3, The Cochrane Collaboration, Oxford, UK). Quality was evaluated using the following seven potential sources of bias: random sequence generation, allocation concealment, blinding of the participants, blinding of outcome assessment, incomplete outcome data, selective outcome reporting, and other sources of bias. The methodology for each RCT was graded as “high,” “low,” or “unclear” to reflect either a high, low, or uncertain risk of bias, respectively.

### Statistical analysis

The statistical analysis was performed using RevMan 5.3. We computed the mean difference (MD) with its 95% confidence intervals (CIs) for continuous variables and the relative risk (RR) with corresponding 95% CIs for dichotomous outcome data. The overall data were determined using a Z-test. All reported *P*-values are two-sided. A two-sided *P*-value < 0.05 was considered to be statistically significant. Statistical heterogeneity was estimated using the *I*^*2*^ statistic, which was deemed to be significant when *I*^2^ values were above 50%. The Mantel-Haenszel or inverse variance fixed-effects model was used for the study without significant heterogeneity, while the Mantel-Haenszel or inverse variance random-effects model was used for the study with significant heterogeneity. Sensitivity analyses were performed by excluding studies with a high risk of bias.

We performed subgroup analyses for primary outcomes on the basis of type of BPB approach (axillary, infraclavicular, interscalene, or supraclavicular), dose of tramadol (50 mg or 100 mg), type of LA (intermediate-acting LA [lidocaine, mepivacaine, or prilocaine] or long-acting LA [ropivacaine, bupivacaine, or levobupivacaine]), and volume of LA used for BPB (≤30 mL or >30 mL).

If the funnel plot was visually asymmetric or if the *P*-values were < 0.1 on Egger’s linear regression test, the presence of a possible publication bias was suspected. In such cases, a trim-and-fill analysis was performed to confirm publication bias.

### Predefined sources of heterogeneity and GRADE guidelines

There was heterogeneity with regard to the definitions of times to onset and duration of sensory block and motor block. Therefore, we assessed the strength of evidence from the RCTs using the GRADE (Grades of Recommendation, Assessment, Development, and Evaluation) guidelines. The GRADE tool evaluates the quality across RCTs for each outcome. Based on key elements, including study quality, consistency, directness, precision, and publication bias, the GRADE tool classifies the strength of the synthesized evidence into four categories: high quality (further research is very unlikely to change the confidence in the estimate of effect); moderate quality (further research is likely to have an important impact on the confidence in the estimate of effect and may change the estimate); low quality (further research is very likely to have an important impact on the confidence in the estimate of effect and is likely to change the estimate); and very low quality (there is a high degree of uncertainty about the estimate).

## Results

### Study search

**Fig 1:**
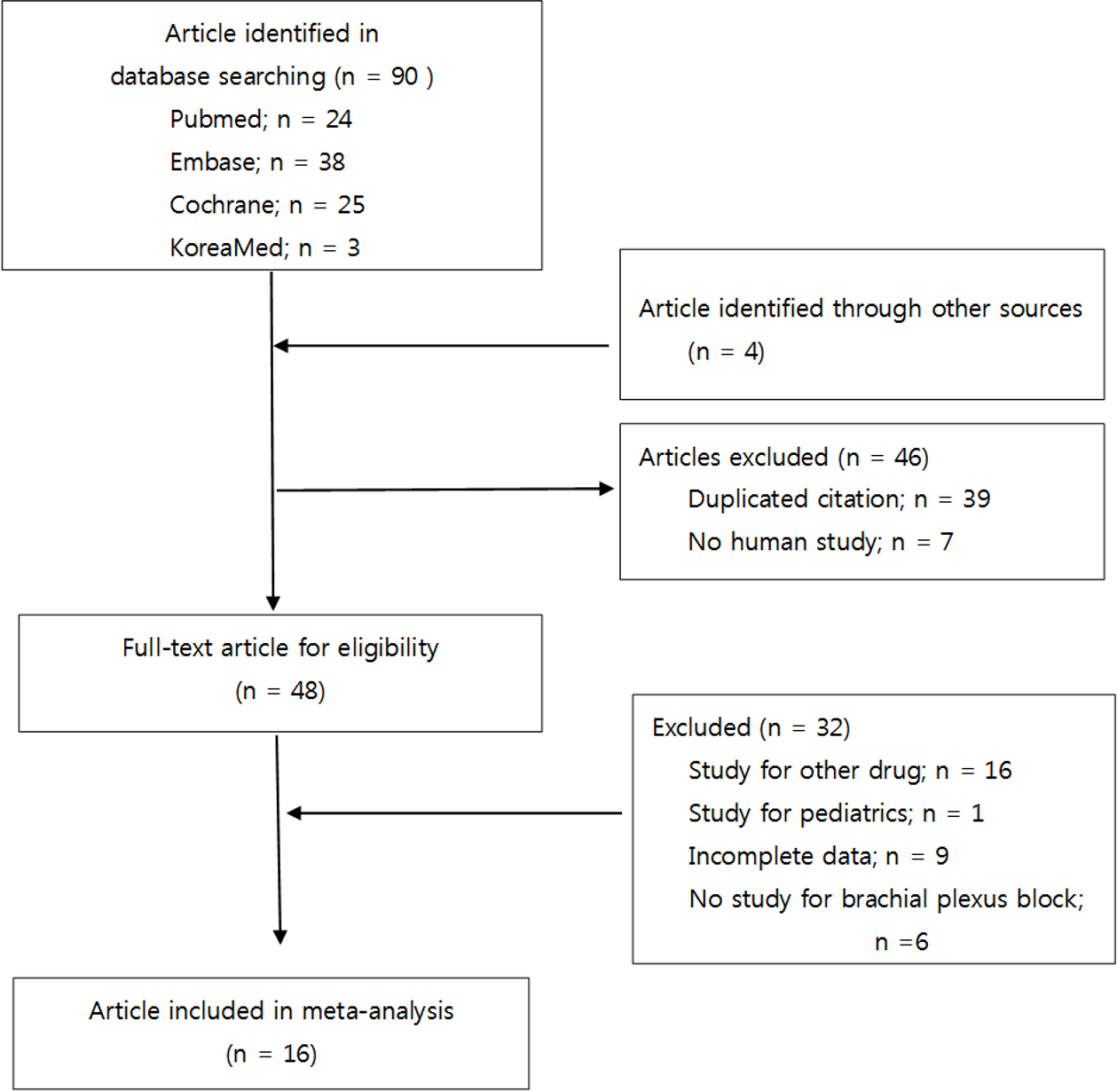
PRISMA flow diagram for the inclusion and exclusion process.

Our initial electronic search identified 94 potential RCTs (24 from PubMed, 38 from EMBASE, 25 from Cochrane CENTRAL, 3 from KoreaMed, and 4 from Google Scholar). We identified 16 studies [16–31] that used tramadol (50 mg or 100 mg) and were published between 1999 and November 2015. These studies included a total of 751 patients (377 who received LA alone and 374 who received LA with tramadol) (Fig 1). No further records were derived from ClinicalTrials.gov or by contacting authors.

### Study characteristics and data

The studies included in this review originated from eight countries, i.e., Austria [31], France [29], Germany [28], India [16, 27], Italy [22, 30], Pakistan [17], Turkey [18, 20, 21, 25, 26], and Tunisia [19, 24]. The patients had undergone various types of surgery, including repair of an arteriovenous fistula [25], carpal tunnel release [29, 30], shoulder arthroplasty [22], and shoulder or upper extremity surgery [16–21, 23, 24, 26–28, 31]. There were no studies using an infraclavicular approach for BPB. The details of BPB were recorded according to type of approach (axillary [17, 18, 20, 21, 24–26, 28–31], interscalene [22], supraclavicular [16, 19, 23, 27]), the technique used for nerve guidance (landmark [16, 17, 23, 27, 28], nerve stimulator [18, 20–22, 24–26, 29–31] or ultrasound guidance [19]), type of LA (bupivacaine [16, 27], levobupivacaine [20, 22], lidocaine [19, 24], mepivacaine [29, 31], prilocaine [28], ropivacaine [18, 23, 25, 26, 30], or a mixture of LA agents [17, 21]), dose of tramadol (50 mg [18, 23], 100 mg, or 1.5 mg/kg [16, 17, 19–22, 24–31]), and the definitions of sensory block, motor block, and analgesia in all the studies (Table 1).

**Table 1:** Characteristics of the included studies.

Reference	Studies	Surgery	Groups	LA volume for BPB	Groups (perineural adjuvant with LA)	Patient age, y	Patients (n)	Characteristics of block		
								Guidance	Type of BPB	Definition of sensory or motor block
**[16]**	Nagpal 2015	Forearm bone fracture surgery	0.5% bupivacaine 18 ml	28	Tramadol 100 mg	20–60	30	Landmark	Supraclavicular	DS–to reappearance of pinprick test using 3-point scale 1, DM–to modified Bromage scale 3, DA–to first rescue analgesic request, OS–to A type pinprick test using 3-point scale 1 (loss of sensation), OM–to modified Bromage scale 0 (motor paralysis of wrist and hand).
					Tramadol 100 mg (IV)		30			
					Control		30			
**[17]**	Khosa 2015	Surgery for forearm and hand	0.5% bupivacaine 20 ml + lidocaine 10 ml with adrenaline	32	Tramadol 100 mg	18–60	30	Landmark	Axillary	DS–using pinprick test response, DM–using Modified Bromage scale, DA–to first rescue analgesic request, OS–to pinprick using 3-point scale, OM–to modified Bromage scale. No clear definitions for DS, DM, DA, OS, and OM.
					Control		30			
**[18]**	Senel 2014	Forearm and hand surgery	0.375% ropivacaine 40 ml	40	Tramadol 50 mg	18–60	12	Nerve stimulator	Axillary	*Sensory and motor check for 4 nerves (radial*, *ulnar*, *median*,*musculocutaneous nerve)*. DS–to reappearance of pinprick test using 3-point scale 1, DM–to modified Bromage scale 3, No clear definitions for DA, OS, OM.
					Control		12			
					Ketamine 50 mg		12			
**[19]**	Trabelsi 2013	Upper limb surgery	2% lidocaine 15 ml	17	Tramadol 100 mg	18–80	20	Ultrasound	Supraclavicular	DS–to reappearance of pinprick test using 3-point scale 1, DM–to modified Bromage scale 3, DA- to first rescue analgesic request, OS–to B type pinprick test using 3-point scale 2 (loss of sensation to touch), OM–to modified Bromage scale 0.
					Control		20			
					Dexamethasone 8 mg		20			
**[20]**	Yurtlu 2012	Hand and forearm surgery	0.5% levobupivacaine 36 ml	38	Tramadol 100 mg	No details given (mean; 36–38)	28	Nerve stimulator	Axillary	*Sensory and motor check for 4 nerves (radial*, *ulnar*, *median*, *musculocutaneous nerve)*. DM–to motor block using 3-point scale 0 (no motor block), DA–to first rescue analgesic request, OS–to loss of sense to B type pinprick using 3-point scale 2 in all 4 nerves, OM–no comment.
					Control		28			
**[21]**	Geze 2012	Hand, forearm, wrist surgery	0.25% levobupivacaine 40 ml + lidocaine 40 mg	40	Tramadol 100 mg	18–60	20	Nerve stimulator	Axillary	*Sensory and motor check for 4 nerves (radial*, *ulnar*, *median*, *musculocutaneous nerve)*. DS–to reappearance of sensory block using 3-point scale 0, DM—to ‘motor block using 3-point scale 0, DA–to first rescue analgesic request, OS—to complete sensory block, OM- to motor block using 3-point scale 0.
					Control		20			
					Fentanyl 50 μg		20			
**[22]**	Alemanno 2012	Shoulder arthroplasty	0.5% levobupivacaine 0.4 ml/kg	24	Tramadol 1.5 mg/kg	Above 18	38	Nerve stimulator	Inter- scalene	DA–to first rescue analgesic request with a VAS > 3.
					Tramadol 1.5 mg/kg (IM)		38			
					Control		39			
**[23]**	Madhusudhana 2011	Upper limb surgery	0.75% ropivacaine	30	Tramadol 50 mg	18–60	10	Landmark	Supra- clavicular	DS–to recovery of sensation, DM, DA–no comments, OS–using pinprick test (complete block), OM- to motor block.
					Control		10			
					Fentanyl 50 μg		10			
**[24]**	Kaabachi 2009	Hand surgery	1.5% lidocaine (1/200,000) 40 ml	30	Tramadol 100 mg	No details given (mean 33–39)	34	Nerve stimulator	Axillary	DS–to recovery of sensory block using 3-point scale 0, DM—to recovery of motor block using 4-point scale 3, DA–to first rescue analgesic request, OS–to loss of sense to B-type pinprick test using 3-point scale 2 (anesthesia).
					Tramadol 200 mg		35			
					Control		33			
**[25]**	Dikmen 2009	Arteriovenous fistula repair	0.375% ropivacaine 38 ml	40	Tramadol 100 mg	30–80	20	Nerve stimulator	Axillary	Uremic patient, DS–to recovery of sensory block using 3-point scale 0, DM–to recovery of motor block using 3-point scale 0 (normal motor function), DA–to first rescue analgesic request, OS–using pinprick test (complete block), OM- to motor block using 3-point scale 2 (complete motor block).
					Control		20			
**[26]**	Kesimici 2007	Hand and forearm surgery	0.75% ropivacaine 40 ml+	42	Tramadol 100 mg	18–65	20	Nerve stimulator	Axillary	*Sensory and motor check for 4 nerves (radial*, *ulnar*, *median*, *musculocutaneous nerve)*, DS–to recovery of sensory block in all 4 nerves, DM–to recovery of motor block, DA–to first rescue analgesic request with VAS score > 4, OS—to loss of sense to B type pinprick using 3-point scale 2 in all 4 nerves, OM–to motor block using 3-point scale 2 (complete motor block) in all 4 nerves.
					Control		20			
**[27]**	Chattopadhyay 2007	Upper limb surgery	0.25% bupivacaine 38 ml + normal saline 2 ml	40	Tramadol 100 mg	18–70	35	Landmark	Supraclavicular	DS–to reappearance of pinprick response, DM–to modified Bromage scale 3, DA–to first rescue analgesic request, No clear definitions for OS, OM.
					Control		35			
**[28]**	Broch 2005	Hand and forearm surgery	1.5% prilocaine 40 ml	40	Tramadol 1.5 mg/kg	Above 18	20	Landmark	Axillary	*Sensory and motor check for 4 nerves (radial*, *ulnar*, *median*, *musculocutaneous nerve)*. DS–to recovery of sensory block in all 4 nerves, DM to recovery of motor block, DA- to first rescue analgesic request.
					Control		20			
**[29]**	Robaux 2004	Carpal tunnel release	1.5% mepivacaine 40 ml	40	Tramadol 40 mg	No details given (mean 45–50)	20	Nerve stimulator	Axillary	DS–to reappearance of pinprick using 3-point scale 2 (normal motor function), DM–to modified Bromage scale 3, OS–to light touch perception using 3-point scale 0 (no sensation).
					Tramadol 100 mg		20			
					Tramadol 200 mg		22			
					Control		17			
**[30]**	Antonucci 2001	Carpal tunnel release	0.75% ropivacaine 20 ml	20	Tramadol 100 mg	23–63	20	Nerve stimulator	Axillary	DS–to recovery of sensory block, DA–full recovery of sense in hands, OS–to B type Pinprick test using 3-point scale 1 (analgesia).
					Control		20			
					Clonidine 1.5 g/kg		20			
					Sufentanil 20 g		20			
**[31]**	Kapral 1999	Forearm and hand surgery	1% mepivacaine 40 ml	40	Tramadol 100 mg	No details given (mean 44–48)	20	Nerve stimulator	Axillary	*Sensory and motor check for 4 nerves (radial*, *ulnar*, *median*, *musculocutaneous nerve)*. DS–to offset of paresthesia, DM–to recovery of motor block.
					Tramadol 100 mg (IV)		20			
					Control		20			

DS, duration of sensory block; DM, duration of motor block; DA, duration of analgesia; OS, onset of sensory block; OM, onset of motor block; A type pinprick test using 3-point scale: 1 = no block (sharp sensation), 2 = partial block (blunt sensation, analgesia), 3 = complete block (no touch sensation, anesthesia). B type pinprick test using 3-point scale: 0 = normal sensation, 1 = loss of sensation of pinprick (analgesia), 2 = loss of sensation of touch (anesthesia). Modified Bromage scale using 4-point scale: 0 = no motion, 1 = finger movement, 2 = wrist flexion, 3 = elbow flexion. Motor block using 3-point scale: 0 = normal motor strength, 1 = reduced motor strength, 2 = complete motor block.

### Risk of bias assessment

**Fig 2:**
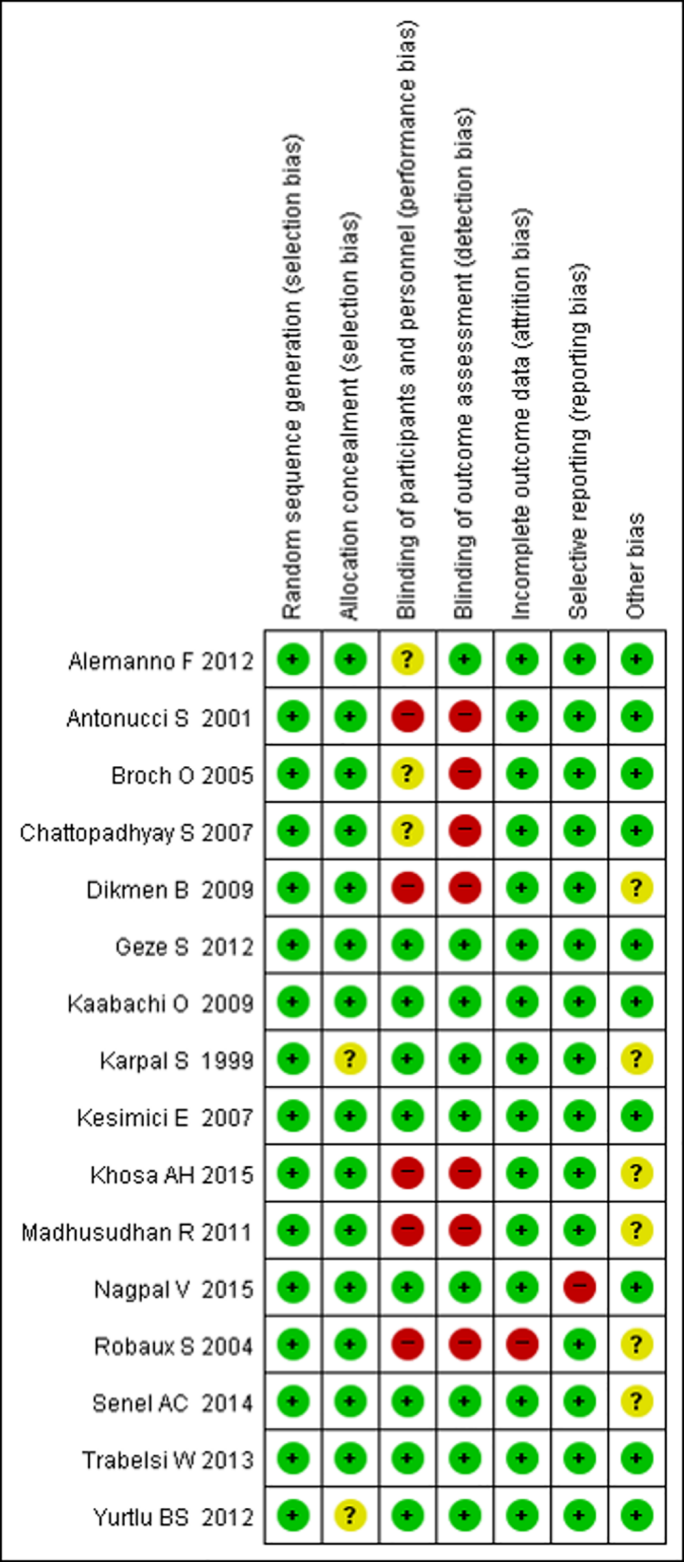
Risk of bias summary for the included studies. Green circle, low risk of bias; yellow circle, unclear risk of bias; red circle, high risk of bias.

A risk of bias assessment was performed to determine study quality and potential bias. All 16 studies mentioned randomization [16–31], and 15 studies included the details of concealed allocation [16–19, 21–31]. However, five studies were conducted without blinding for assessment of outcomes [17, 23, 25, 30, 31]. One study did not state the details of exclusion in the number in each group [29] and the other study reported selective outcomes [16] (Fig 2).

### Publication bias

We evaluated a funnel plot for every comparison and estimated the publication bias using Egger’s linear regression method. Egger’s linear regression method indicated the publication bias for the following comparisons (>10 studies for comparison): duration of sensory block (*P* = 0.00985), duration of motor block (*P* = 0.01386), duration of analgesia (*P* = 0.00995), and time to onset of sensory block (*P* = 0.069381). However, no publication bias was noted for the time to onset of motor block (*P* = 0.5354). To compare *P*-values < 0.1 derived by Egger’s method, we performed a trim-and-fill analysis, and noticed a change in the significance of the results for the time to onset of sensory block (95% CI, -0.55 to 1.66). However, we noted no changes in the statistical significance of the results for duration of sensory block, motor block, and analgesia, indicating publication bias for these three parameters.

### Results of the meta-analysis

#### 1. Duration of sensory block [16–19, 21, 23–31]

**Fig 3:**
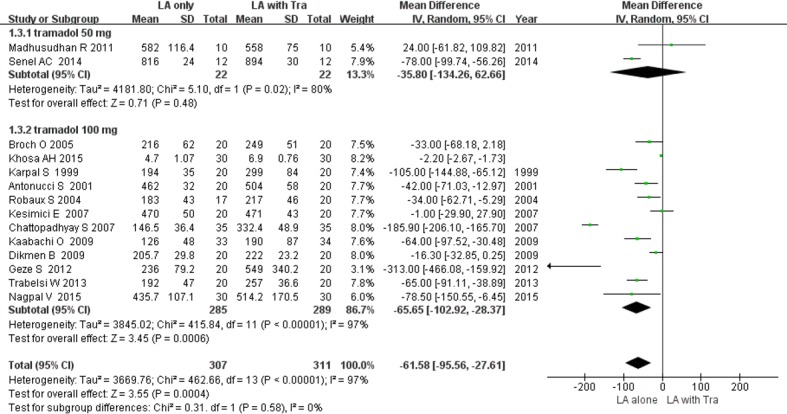
Forest plot demonstrating the duration of sensory block. Subgroup analysis according to dose of tramadol. CI, confidence interval; LA, local anesthesia; SD, standard deviation; Tra, tramadol.

The duration of sensory block was defined using the pinprick test [16–19, 21, 24, 25, 27, 29], recovery of sensation [23, 26, 28, 30], and offset of paresthesia [30] (Table 1). Adjuvant use of tramadol significantly prolonged the duration of sensory block by 61.5 min, with high heterogeneity (14 RCTs; 95% CI, -95.5 to -27.6; I^2^ = 97%; *P* = 0.0004) (Fig 3). In subgroup analysis of the BPB approach, the duration of sensory block was prolonged in the studies with axillary approach (MD, -45.6 min; *P* = 0.0002), but not in the studies with interscalene or supraclavicular approach (MD, -81.7 min; *P* = 0.07; Table 2) (S1 Table). In subgroup analysis of the tramadol dose, the duration of sensory block was prolonged in the studies with tramadol 100 mg (MD, -65.6 min; *P* = 0.0006), but not in the studies with tramadol 50 mg (MD, -35.8 min; *P* = 0.48; Fig 3). Sensitivity analysis did not detect any change in the overall significance of the duration of sensory block.

**Table 2:** Subgroup meta-analysis by type of BPB approach.

	Interscalene or supraclavicular approach				Axillary approach				Subgroup differences		Test for overall effect *P*
	Stud ies (n)	MD (95% CI)	*I*^*2*^	*P*	Studies (n)	MD (95% CI)	*I*^*2*^	*P*			
									*I*^*2*^	*P*	
**Duration of sensory block**	4	-81.7 (-169.7, 6.3)	96%	0.07	10	-45.6 (-69.9, -27.6)	92%	0.0002	0%	0.44	0.0004
**Duration of motor block**	4	-88.9 (-152.5, -25.4)	86%	0.006	10	-54.9 (-92.1, -17.8)	97%	0.004	0%	0.37	0.0003
**Duration of analgesia**	5	-147.6 (-255.4, -39.8)	94%	0.007	9	-107.7 (-165.0, -50.5)	98%	0.0002	0%	0.52	< 0.00001

A *P* value < 0.05 was considered statistically significant. BPB, brachial plexus block; CI, confidence interval; *I*^2^, statistic for heterogeneity; LA, local anesthesia; MD, mean difference (min). No studies using an infraclavicular approach were identified in the literature.

#### 2. Duration of motor block [16–21, 23–29, 31]

**Fig 4:**
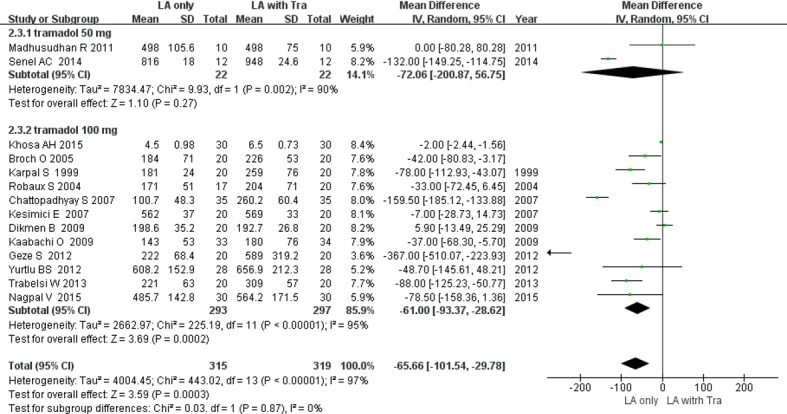
Forest plot demonstrating the duration of motor block. Subgroup analysis according to dose of tramadol. CI, confidence interval; LA, local anesthesia; SD, standard deviation; Tra, tramadol.

The duration of motor block was defined using the modified Bromage scale [16–19, 27, 29], a 3-point scale [20, 21, 25], a 4-point scale [24], or recovery of motor block [26, 28, 31], as shown in Table 1. Use of tramadol as an adjuvant prolonged the duration of motor block by 65.6 min, with high heterogeneity (14 RCTs; 95% CI, -101.5 to -29.7; *I*^2^ = 97%; *P* = 0.0003; Fig 4). In subgroup analysis, the duration was prolonged in the studies with tramadol 100 mg (MD, -61.0 min; *P* = 0.0002), but not in the studies with tramadol 50 mg (MD, -72.0 min; *P* = 0.27; Fig 4) (S1 Table). Sensitivity analysis did not reveal any change in the overall significance of the duration of sensory block.

#### 3. Duration of analgesia [16–28, 30]

**Fig 5:**
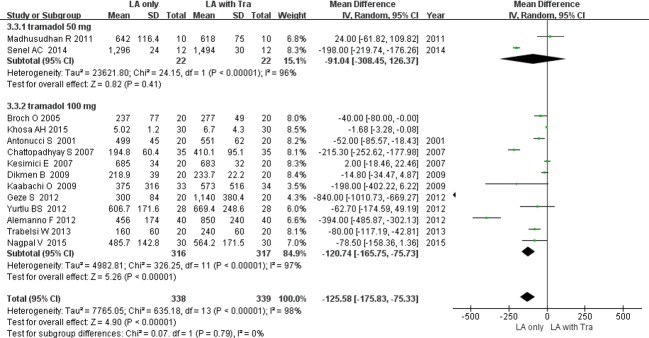
Forest plot demonstrating the duration of analgesia. Subgroup analysis by dose of tramadol. CI, confidence interval; LA, local anesthesia; SD, standard deviation; Tra, tramadol.

The duration of analgesia was defined as the time to first request for rescue analgesia [16, 17, 19–21, 24, 25, 27, 28], time to first request for rescue analgesia with a visual analog scale score >3 [22], or time to first request for rescue analgesia with a visual analog scale score >4 [26] (Table 1). Use of tramadol as an adjuvant significantly prolonged the duration of analgesia by 125.5 min with high heterogeneity (14 RCTs; 95% CI, -175.8 to -75.3; *I*^2^ = 98%; *P* < 0.0001; (Fig 5). In subgroup analysis, the duration was prolonged in the studies with tramadol 100 mg (MD, -120.7 min; *P*< 0.000001), but not in the studies with tramadol 50 mg (MD, -91.0 min; *P* = 0.41; Fig 5) (S1 Table). Sensitivity analysis did not reveal any change in the overall significance of the duration of analgesia.

#### 4. Time to onset of sensory block [16–21, 23–27, 29, 30]

**Fig 6:**
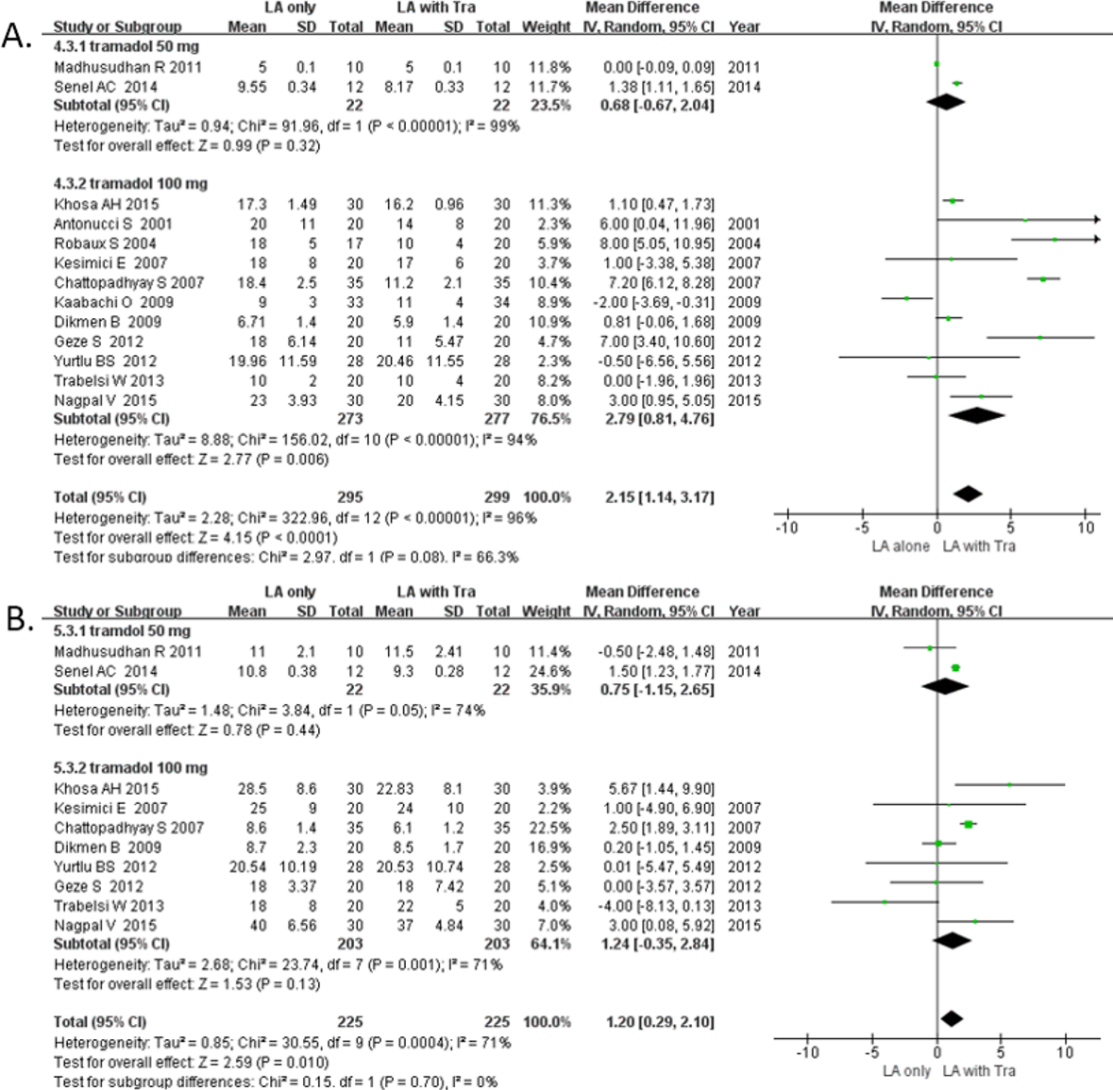
**Forest plot demonstrating (A) time to onset of sensory block and (B) time to onset of motor block.** Subgroup analysis by dose of tramadol. CI, confidence interval; LA, local anesthesia; SD, standard deviation; Tra, tramadol.

The time to onset of sensory block was defined using the pinprick test using a 3-point scale (A type [16]: 1 = no block [sharp sensation], 2 = partial block [blunt sensation, analgesia], 3 = complete block [no touch sensation, anesthesia]; B type [16, 20, 24, 26, 30]: 0 = normal sensation, 1 = loss of sensation of pinprick [analgesia], 2 = loss of sensation of touch [anesthesia]), complete sensory block [21, 23], or light touch perception using a 3-point scale [29] (Table 1). Adjuvant use of tramadol shortened the time to onset of sensory block by 2.1 min, with high heterogeneity (13 RCTs; 95% CI, 1.1 to 3.1; *I*^2^ = 96%; *P* < 0.0001; Fig 6A). Sensitivity analysis did not detect any change in the overall significance of the time to onset of sensory block.

#### 5. Time to onset of motor block [16–21, 23, 25–27]

The time to onset of motor block was determined using the modified Bromage scale [16–19], a 3-point scale [20, 25, 26], or as complete motor block [21, 23] (Table 1). Adjuvant use of tramadol shortened the time to onset of motor block by 1.20 min with high heterogeneity (10 RCTs; 95% CI, 0.2 to 2.1; *I*^2^ = 71%; *P* = 0.010; Fig 6B). Sensitivity analysis did not detect any change in the overall significance of the time of motor block.

#### 6. Adverse effects

Tramadol use did not change the incidence of adverse effects after BPB between the study groups: nausea (10 RCTs; RR, 0.61; 95% CI, 0.29 to 1.30; I^2^ = 0%; *P* = 0.92; number needed to treat (NNT) = 22) [16–18, 20–22, 25, 26, 29, 31], vomiting (10 RCTs; RR, 0.76; 95% CI, 0.30 to 1.93; I^2^ = 0%; *P* = 0.97; NNT = 39) [17, 18, 20–22, 25–27, 29, 31], pruritus (5 RCTs; RR, 0.23; 95% CI, 0.04 to 2.00; I^2^ = 0%; *P* = 0.98; NNT = 30) [17, 20, 21, 26, 29], and sedation (4 RCTs; RR, 0.60; 95% CI, 0.16 to 2.29; I^2^ = 0%; *P* = 0.93; NNT = 32) [16–18, 29] (Table 3).

**Table 3:** Incidence of adverse effects of tramadol.

Adverse effects	Number of tramadol/Total number of patients		RR (95% CI)	*P*	NNT	Reference
	LA only	LA with tramadol				
Nausea	225/453	228/453	0.61 (0.29 to 1.30)	0.61	22	[16–18, 20–22, 25, 26, 29, 31]
Vomiting	230/463	233/463	0.76 (0.30 to 1.93)	0.34	39	[17, 18, 20–22, 25–27, 29, 31]
Pruritus	115/233	118/233	0.23 (0.04 to 2.00)	0.18	30	[17, 20, 21, 26, 29]
Sedation	89/121	92/121	0.60 (0.16 to 2.29)	0.42	32	[16–18, 29]

CI, confidence interval; LA, local anesthesia; NNT, number needed to treat; RR, risk ratio

#### 7. GRADE guidelines

In subgroup analysis according to tramadol dose, the duration of sensory block, motor block, and analgesia was prolonged in the studies with tramadol 100 mg for BPB but not in the studies with tramadol 50 mg. When the strength of the evidence was evaluated using the GRADE guidelines, there was high evidence that tramadol 100 mg with LA for BPB prolonged the duration of analgesia when compared with LA alone for BPB in patients undergoing upper extremity surgery (Table 4). The overall quality assessment was downgraded by inconsistency of effect, heterogeneity, and publication bias, but upgraded by the larger treatment effect and the presence of a dose-response relationship.

**Table 4:** Effect of tramadol as an adjuvant to local anesthesia according to tramadol dose (50 mg or 100 mg) for brachial plexus block.

Outcomes	Illustrative comparative risks* (95% CI)		Participants (studies)	Quality of evidence (GRADE)	Comments
	LA alone for BPB (control)	LA with tramadol for BPB (intervention)			Test of overall effect(P)
Duration of sensory block–tramadol 50 mg	Mean duration of sensory block–LA alone in the control group was 699.0 min	Mean duration of sensory block -LA with tramadol 50 mg in the intervention groups was **35.8 min longer** (-134.26 longer)	44 (2 studies)	⊕⊕⊝⊝ ^¶^ **low**	*P =* 0.48 (not statistically significant)
Duration of sensory block- tramadol 100 mg	Mean duration of sensory block–LA alone in the control group was 239.3 min	Mean duration of sensory block -LA with tramadol 100 mg in the intervention groups was **65.6 min longer** (28.37–102.92 longer)	574 (12 studies)	⊕⊕⊕⊝ ^&^* moderate	*P* = 0.0006
Duration of motor block- tramadol 50 mg	Mean duration of sensory block–LA alone in the control group was 657.0 min.	Mean duration of motor block–LA with tramadol 50 mg in the intervention groups was **72.0 min longer** (- 200.87 longer)	44 (2 studies)	⊕⊕⊝⊝ ^¶^ **low**	*P =* 0.27 (not statistically significant)
Duration of motor block–tramadol 100 mg	The mean duration of sensory block–LA alone in the control group was 256.8 min.	Mean duration of motor block–LA with tramadol 100 mg in the intervention groups was **61.0 min longer** (28.62–93.37 longer)	590 (12 studies)	⊕⊕⊕⊝ ^&^* moderate	*P =* 0.0002
Duration of analgesia–tramadol 50 mg	Mean duration of sensory block–LA alone in the control group was 969 min.	Mean duration of analgesia–LA with tramadol 50 mg in the intervention groups was **91.0 min longer** (-308.45 longer)	44 (2 studies)	⊕⊝⊝⊝ ^¶^^&^ **very low**	*P =* 0.41 (not statistically significant)
Duration of analgesia–tramadol 100 mg	Mean duration of sensory block–LA alone in the control group was 351.9 min.	Mean duration of analgesia–LA with tramadol 100 mg in the intervention groups was **120.7 min longer** (75.73–165.75 longer)	633 (12 studies)	⊕⊕⊕⊕^&^* ^#a^ **high**	*P* < 0.00001
Onset of sensory block–tramadol 50 mg	Mean duration of sensory block–LA alone in the control group was 7.2 min.	Mean onset of sensory block–LA with tramadol 50 mg in the intervention groups was **0.68 min shorter** (-2.04 shorter)	44 (2 studies)	⊕⊕⊝⊝ ^&^ **low**	*P =* 0.32 (not statistically significant)
Onset of sensory block–tramadol 100 mg	Mean duration of sensory block–LA alone in the control group was 16.2 min.	Mean onset of sensory block–LA with tramadol 100 mg in the intervention groups was **2.79 min shorter** (0.81–4.76 shorter)	550 (11 studies)	⊕⊕⊕⊝ ^¶^ **moderate**	*P =* 0.006
Onset of motor block–tramadol 50 mg	Mean duration of sensory block–LA alone in the control group was 10.9 min.	Mean onset of motor block–LA with tramadol 50 mg in the intervention groups was **0.75 min shorter** (- 2.65 shorter)	44 (2 studies)	⊕⊕⊝⊝ ^¶^ **low**	*P =* 0.44 (not statistically significant)
Onset of motor block–tramadol 100 mg	Mean duration of sensory block–LA alone in the control group was 20.9 min.	Mean onset of motor block–LA with tramadol 100 mg in the intervention groups was **1.24 min shorter** (- 2.84 shorter)	406 (8 studies)	⊕⊕⊝⊝ ^¶^ **low**	*P =* 0.13 (not statistically significant)

GRADE Working Group grades of evidence. High quality: further research is very unlikely to change confidence in the estimate of effect. Moderate quality: further research is likely to have an important impact on confidence in the estimate of effect and may change the estimate. Low quality: further research is very likely to have an important impact on confidence in the estimate of effect and is likely to change the estimate. Very low quality: high degree of uncertainty about the estimate.

^¶^Rated down because of inconsistency of effect.

^&^Rated down because of wide 95% CI with significant heterogeneity (*I*^2^ >95%).

*Rated down by publication bias.

^#a^Rated up by evidence of a large effect and a dose-response relationship.

BPB, brachial plexus block; CI, confidence interval; LA, local anesthesia

## Discussion

Our systemic review and meta-analysis indicates that use of tramadol as an adjuvant to LA in BPB prolongs the duration of sensory block, motor block, and analgesia and that it shortens the time to onset of sensory block and motor block without any change in adverse effects. There was some heterogeneity between the studies with regard to definitions of analgesia, sensory block, and motor block. There was high evidence according to GRADE guidelines that tramadol 100 mg with LA for BPB prolonged the duration of analgesia when compared with LA alone for BPB. To our knowledge, this is the first systematic review to evaluate the effect of tramadol as an adjuvant to LA in BPB for shoulder and upper extremity surgery.

In the past, there have been contradictory results regarding the effect of opioids as an adjuvant to LA in BPB. Saryazdi et al. [7] reported that addition of different opioids (meperidine, buprenorphine, morphine, and fentanyl) to lidocaine in axillary BPB achieved no statistically significant difference in duration of sensory block or motor block between the study groups.

Tramadol has unique modes of action, including weak opioid activity via the μ receptor, α_2_-adrenergic and serotonergic agonistic activity, and LA properties via blockade of K^+^ channels [33–35].

Our study included 16 studies that examined the effect of tramadol as an adjuvant to LA for BPB and also included quality control. However, the studies included in the review showed high heterogeneity. Generally, the type of surgery performed often determines the selection of BPB approach (interscalene, supraclavicular, infraclavicular, or axillary). This can affect the duration of analgesia at the surgical site. As an example, interscalene approaches are used for shoulder surgery, whereas axillary approaches tend to be used more for surgery on the forearm and hand. This difference in approach contributes to different results and clinical heterogeneity. We performed the meta-analysis using RevMan statistical software and performed subgroup analysis for various items (type of BPB approach, dose of tramadol, type of LA, volume of LA used for BPB) to identify the source of the heterogeneity (S1 Table). We could not find any difference in the duration of sensory, motor block, or analgesia according to type of BPB approach, but we did identify a dose-response effect of tramadol (50 mg, 100 mg) on the duration of sensory block, motor block, and analgesia.

Tramadol as an adjuvant for BPB in our review shortened the time to onset of sensory block and motor block. These findings are attributed to the potentiating effect of opioids and the peripheral LA-like effect of tramadol. The mechanism underlying the LA effect of tramadol is different from that of LA; the action of LA is generated by blocking Na^+^ channels, but tramadol exerts its effect by blocking K^+^ channels, as does meperidine [34]. A previous study showed that tramadol was as effective as lidocaine when injected subcutaneously in patients undergoing minor superficial procedures [36]. For the variable route of tramadol during BPB with LA, sensory and motor blocks enhanced by a perineural adjuvant to LA, but not by systemic administration (31).

Typical adverse effects of tramadol are headache, nausea, vomiting, dizziness, and sedation when it is used for analgesia (10, 31). We could not detect any differences in adverse effects between studies in our meta-analysis, which could reflect low plasma concentrations of tramadol. Use of tramadol as an adjuvant in BPB causes fewer symptoms than does intravenous administration of tramadol (36). There have been no reports of nerve damage attributed to tramadol in animal or human studies. The US Food and Drug Administration has not approved perineural administration of tramadol as it has for dexamethasone.

A recent systematic review of various adjuvants for peripheral nerve block [36] reported results for tramadol that contradict the findings of our systematic review. The authors of that review reported that perineural tramadol had no effect on sensory or motor block, and recommended not using tramadol as an adjuvant in peripheral nerve block. However, their review included only 5 RCTs of tramadol as an adjuvant to LA in BPB [22, 24, 26, 29, 31], and omitted many other relevant RCTs [16–20, 23, 25, 28]. Furthermore, they also included RCTs for other types of nerve block, such as psoas block [37] and paravertebral block [38]. Unlike that review of tramadol, we systematically searched for and identified the 16 studies on tramadol used as an adjuvant alone in BPB [16–31], and analyzed the effects of tramadol on sensory block, motor block, and analgesia using systemic meta-analysis software. Generally, the degree of nerve block is determined by the type of nerve, the anatomic site of the nerve, and the type of nerve block.

Our review has several limitations. First, the studies included in the review contained considerably clinical heterogeneity with regard to type of BPB approach, dose and volume of drug, and type of guidance used for BPB. Based on the clinical assumption that different types of BPB may lead to different sensory or motor block characteristics and analgesia. Second, the definitions of outcomes of interest such as time to onset and duration of sensory block, motor block, and analgesia varied widely between the studies. Third, this review pertains to the duration of sensory block, motor block, and analgesia, and highlighted publication bias as ascertained by the trim-and-fill analysis. As a result, the findings of our meta-analysis were influenced by publication bias among the included studies.

However, our review also has several strengths. The main strength is that we tried to include all relevant databases and RCTs in our search. The methodology used was strong, with registration of the protocol for the review on PROSPERO and use of RevMan software.

## Conclusions

Our study provides evidence that tramadol 100 mg is a potential adjuvant for use with LA in BPB. Adjuvant tramadol prolonged the duration of sensory block, motor block, and analgesia and shortened the time to onset of sensory block and motor block without altering the incidence of adverse effects.

## Supporting information

S1 File The search strategy. (DOCX)

S1 Table Summary of subgroup analysis from the results of meta-analysis. (DOCX)

S2 Table The PRISMA checklist. (DOC)
