# Supplementary material for: Effect of tramadol as an adjuvant to local anesthetics for brachial plexus block: A systematic review and meta-analysis
Source: PLoS One. 2017 Sep 27;12(9):e0184649. doi: 10.1371/journal.pone.0184649 (PMC5617157; doi:10.1371/journal.pone.0184649)
Supplement: S1 File — (DOCX) [file pone.0184649.s001.docx]

# **S1 File. The search strategy**

**Pubmed**

1. Search brachial plexus block[mesh]

2. Search brachial plexus block[tiab] OR brachial plexus blocks[tiab] OR brachial plexus anesthesia[tiab] OR brachial plexus blockade[tiab] OR brachial plexus blockades[tiab]

3. Search (#1 OR #2)

4. Search brachial plexus [mesh] AND nerve block[mesh]

5. Search brachial plexus[tiab] AND (nerve block[tiab] OR nerve blocks[tiab] OR nerve blockade[tiab] OR nerve blockades[tiab])

6. Search (#4 OR #5)

7. Search axillary block [tiab] OR axillary blocks [tiab] OR infraclavicular block[tiab] OR infraclavicular blocks[tiab] OR interscalene block[tiab] OR interscalene blocks[tiab] OR supraclavicular block[tiab] OR supraclavicular blocks[tiab]

8. Search (#3 OR #6 OR #7)

9. Search Local Anesthetics[mesh]

10. Search Local Anesthetics[tiab] OR Local Anesthetic[tiab]

11. Search levobupivacaine[tw] OR lidocaine[tw] OR bupivacaine[tw]

12. Search (#9 OR #10 OR #11)

13. Search tramadol[mesh] OR tramadol[tiab]

14. Search (#8 AND #12 AND #13)

**EMBASE** ( < 1966 – 2015 publication year)

1. 'brachial plexus anesthesia'/exp

2. 'brachial plexus block':ti,ab OR 'brachial plexus blocks':ti,ab OR 'brachial plexus anesthesia':ti,ab OR 'brachial plexus blockade':ti,ab OR 'brachial plexus blockades':ti,ab

3. 'brachial plexus anesthesia'/exp OR 'brachial plexus block':ti,ab OR 'brachial plexus blocks':ti,ab OR 'brachial plexus anesthesia':ti,ab OR 'brachial plexus blockade':ti,ab OR 'brachial plexus blockades':ti,ab

4. 'brachial plexus'/exp AND 'nerve block'/exp

5. 'brachial plexus':ti,ab AND ('nerve block':ti,ab OR 'nerve blocks':ti,ab OR 'nerve blockade':ti,ab OR 'nerve blockades':ti,ab)

6. 'brachial plexus'/exp AND 'nerve block'/exp OR ('brachial plexus':ti,ab AND ('nerve block':ti,ab OR 'nerve blocks':ti,ab OR 'nerve blockade':ti,ab OR 'nerve blockades':ti,ab))

7. 'axillary block':ti,ab OR 'axillary blocks':ti,ab OR 'infraclavicular block':ti,ab OR 'infraclavicular blocks':ti,ab OR 'interscalene block':ti,ab OR 'interscalene blocks':ti,ab OR 'supraclavicular block':ti,ab OR 'supraclavicular blocks':ti,ab

8. 1 OR 2 OR 3 OR 4 OR 5 OR 6 OR 7

9. 'local anesthetic agent'/exp

10. 'local anesthetics':ti,ab OR 'local anesthetic':ti,ab

11. 'levobupivacaine':ti,ab OR 'lidocaine':ti,ab OR 'bupivacaine':ti,ab

12. 'levobupivacaine'/exp OR 'lidocaine'/exp OR 'bupivacaine'/exp

13. 9 OR 10 OR 11 OR 12

14. 'tramadol'/exp OR tramadol:ti,ab

15. [controlled clinical trial]/lim OR [randomized controlled trial]/lim

16. 8 AND 13 AND 14 AND 15

**Cochrane** (publication year 2015)

1. [mh " Brachial Plexus Block"]

2. ("brachial plexus block" or "brachial plexus blocks" or "brachial plexus anesthesia" or "brachial plexus blockade" or "brachial plexus blockades"):ti,ab

3. #1 or #2

4. [mh "brachial plexus"]

5. [mh "nerve block"]

6. #4 and #5

7. "brachial plexus":ti,ab and ("nerve block" or "nerve blocks" or "nerve blockade" or "nerve blockades"):ti,ab

8. #6 or #7

9. ("axillary block" or "axillary blocks" or "infraclavicular block" or "infraclavicular blocks" or "interscalene block" or "interscalene blocks" or "supraclavicular block" or "supraclavicular blocks"):ti,ab 2

#10 #3 or #8 or #9

#11 [mh "Local Anesthetics"]

#12 ("Local Anesthetics" or "Local Anesthetic"):ti,ab

#13 [mh levobupivacaine] or [mh lidocaine] or [mh bupivacaine]

#14 (levobupivacaine or lidocaine or bupivacaine):ti,ab

#15 {or #11-#14}

#16 [mh tramadol] or tramadol:ti,ab

#17 #10 and #15 and #16

Search No. (Duplication No.)

PubMed 24 (0)

EMBASE 38 (12)

Cochrane 25 (23)

Koreamed 3 (0)

Total 90 (35)
