# Supplementary material for: Effect of tramadol as an adjuvant to local anesthetics for brachial plexus block: A systematic review and meta-analysis
Source: PLoS One. 2017 Sep 27;12(9):e0184649. doi: 10.1371/journal.pone.0184649 (PMC5617157; doi:10.1371/journal.pone.0184649)
Supplement: S1 Table — (DOCX) [file pone.0184649.s002.docx]

**S1 Table.** **Summary of Subgroup Analysis from the Results of Meta-Analysis.**

|  | **Type of BPB approach** | | **Tramadol dose** | | **Type of LA** | | **Volume of LA** | | **Test of overall effect**  ***(P)*** |
| --- | --- | --- | --- | --- | --- | --- | --- | --- | --- |
|  | **interscalene or supraclavicular** | **axillary** | **50 mg** | **100 mg** | **Intermedi ate-acting LA** | **long-acting LA** | **≤ 30 ml** | **> 30 ml** |  |
| **Duration of sensory block** | 0.07 | 0.0002 | 0.48 | 0.0006 | < 0.0001 | 0.01 | < 0.00001 | 0.0002 | 0.0004 |
| **Duration of motor block** | 0.006 | 0.004 | 0.27 | 0.0002 | < 0.0001 | 0.03 | 0.01 | 0.0008 | 0.0003 |
| **Duration of analgesia** | 0.007 | 0.0002 | 0.41 | < 0.00001 | < 0.0001 | 0.005 | 0.001 | < 0.0001 | < 0.0001 |

Subgroup; type of BPB approach (interscalene, and supraclavicular or axillary), dose of tramadol (50 mg or 100 mg), type of LA (intermediate-acting LA [lidocaine, mepivacaine, or prilocaine] or long-acting LA [ropivacaine, bupivacaine, or levobupivacaine]), and volume of LA used for BPB (≤ 30 ml or > 30 ml). A *P* value < 0.05 was considered statistically significant. LA, local anesthetics; BPB, brachial plexus block. There were no studies for infraclavicular approach in the literature after searching process.
